# Genetic Variants in *MTHFR* Gene Predict ≥ 2 Radiation Pneumonitis in Esophageal Squamous Cell Carcinoma Patients Treated with Thoracic Radiotherapy

**DOI:** 10.1371/journal.pone.0169147

**Published:** 2017-01-03

**Authors:** Yang Zhang, Zongjuan Li, Jian Zhang, Hongsheng Li, Yumei Qiao, Chengsuo Huang, Baosheng Li

**Affiliations:** 1 School of Medicine and Life Sciences, University of Jinan-Shandong Academy of Medical Sciences, Jinan, Shandong province, China; 2 Department of Radiation Oncology, Shandong Cancer Hospital Affiliated to Shandong University, Shandong Academy of Medical Sciences, Jinan, Shandong province, China; 3 Department of Radiation Oncology, The Second Hospital of Dalian Medical University Dalian, Liaoning province, China; 4 Department of Ophthalmology and Otorhinolaryngology, The Sixth People’s Hospital of Jinan, Jinan, Shandong province, China; 5 Department of Internal Medicine, Shandong Cancer Hospital Affiliated to Shandong University, Shandong Academy of Medical Sciences, Jinan, Shandong province, China; Duke Cancer Institute, UNITED STATES

## Abstract

Reactive oxygen species (ROS), formed as an indirect production of radiotherapy (RT), could cause DNA damage of normal tissues. Meanwhile, our body possesses the ability to restore the damage by DNA repair pathways. The imbalance between the two systems could finally result in radiation injury. Therefore, in this prospective cohort study, we explored the association of genetic variants in ROS metabolism and DNA repair pathway-related genes with radiation pneumonitis (RP). A total of 265 locally advanced esophageal squamous cell carcinoma (ESCC) patients receiving RT in Chinese Han population were enrolled. Five functional single nucleotide polymorphisms (SNPs) (rs1695 in *GSTP1*; rs4880 in *SOD2*; rs3957356 in *GSTA1*; and rs1801131, rs1801133 in *MTHFR*) were genotyped using the MassArray system, and rs1801131 was found to be a predictor of ≥ 2 RP. Our results showed that, compared with TT genotype, patients with GG/GT genotypes of rs1801131 had a notably lower risk of developing ≥ 2 RP (HR = 0.339, 95% CI = 0.137–0.839, P = 0.019). Further independent studies are required to confirm this findings.

## Introduction

Esophageal carcinoma was diagnosed in nearly 477,900 individuals in China alone in 2015 [1]. Esophageal squamous cell carcinoma (ESCC) represents approximately 95% of all cases of esophageal cancer in the Chinese population. Radiotherapy (RT), alone or in combination with chemotherapy is the mainstay for the management of locally advanced ESCC. Higher doses of RT are closely associated with improved local control; however, radiation pneumonitis (RP) remains a significant barrier to elevated radiation doses and can substantially compromise the quality of life.

Previous studies have confirmed that multiple treatment-related and patient-related factors, such as dosimetric parameters, chemotherapy agents, age, pulmonary function, smoking status, and sex are correlated with RP [2–4]. Unfortunately, these factors are insufficient to explain interindividual variability [5]. Therefore, identification and application of new biomarkers, together with traditional dosimetric and clinical determinants of RP, could significantly improve the tailoring of RT treatment [6].

Single nucleotide polymorphisms (SNPs), recognized as biological genetic markers, have been reported to participate in many biological processes. Recent studies have also identified SNPs of candidate genes involved in DNA damage repair, pro-inflammatory responses, and oxidative stress are potential biomarkers for RP [7–9].

RT causes cytotoxicity through direct ionization of normal tissues and a combination of free radicals, such as reactive oxygen species (ROS) formed by radiolysis of water [10]. These ROS, reacting with biomacromolecules, can damage DNA, resulting in cell death [11–13]. The enzymes that play a key role in ROS metabolism are manganese superoxide dismutase (SOD2) and glutathione S-transferases (GSTs, alpha, pi). The former, enables the dismutation of superoxide radicals to hydrogen peroxide and oxygen [14–15].The latter has the potential to neutralize ROS [16]. Previous study revealed that single nucleotide polymorphisms of genes in ROS metabolism pathway are closely associated with acute skin toxicity for breast cancer patients receiving radiotherapy [10]. So it is highly plausible these genetic variants could also affect radiation lung injury, however, which has not well been explored.

Meanwhile, our body possesses the ability to restore the damage by DNA repair pathways. *MTHFR* is one of the most significant genes involving in that path. One article reported that single nucleotide polymorphisms in *MTHFR* gene could exert influence on the incidence of radiation pneumonitis in white lung cancer patients [17]. As we know, genetic variants may have racial differences, it is unknown whether this effect still exist in our Chinese Han population. Thus, it still possesses significance to further validate this finding.

In view of the above mentioned, we therefore depended on the existing literature by screening for an association of functional SNPs in *SOD2*, *GSTP1*, *GSTA1*and *MTHFR* genes with the risk of RP in a prospective cohort of ESCC patients treated with definitive RT.

## Materials and Methods

### Ethics statement

This study was approved by the Institutional Review Board of Shandong Cancer Hospital Affiliated to Shandong University and informed consents were signed before treatment. We complied with HIPAA regulations.

### Patient population

For our prospective cohort study, a total of 312 ESCC patients receiving definitive thoracic RT between October 2014 and January 2016 from Shandong Cancer Hospital Affiliated to Shandong University, were initially enrolled. Patients with pathologically diagnosed Ⅱ-Ⅳ (American Joint Committee On Cancer, 7th edition) ESCC, a Karnofsky performance status (KPS) of >60, and a life expectancy of at least 6 months were included. After we excluded those who had previously undergone thoracic radiation or esophagectomy, and those who were lost to follow up, the remaining 265 patients formed the final study cohort. Detailed clinical information and dosimetry parameters were also collected.

### Follow-up and evaluation of RP

All patients enrolled in this study were examined during RT and at 1 month after completing RT. Patients were then followed up every 3 months. Radiographic examination by chest X-ray or computerized tomography was performed at each follow-up visit after completion of treatment. RP was independently graded by three radiation oncologists according to Common Terminology Criteria for Adverse Events 4.0, (CTCAE 4.0) as follows: Grade 0, no change; Grade 1, asymptomatic and diagnosed by radiographic findings only; Grade 2, symptomatic, not interfering with daily activities; Grade 3, symptomatic, interfering with daily activities or oxygen required; Grade 4, assisted ventilation required; Grade 5, fatal.

### SNP selection and genotyping

A 5ml whole-blood sample was obtained from each patients before RT, and genomic DNA was isolated from the whole-blood samples using AxyPrep Blood Genomic DNA Miniprep Kit (Axygen, USA). Genotyping was performed by Sequenom MassArray System (San Diego, USA) on known functional SNPs for candidate genes in ROS metabolism and DNA repair pathways including: 1) *glutathione S-transferases pi 1* (*GSTP1*; rs1695); 2) *superoxide dismutase 2* (*SOD2*; rs4880); 3) *glutathione S-transferases alpha 1*(*GSTA1*; rs3957356); 4) *methylenetetrahydrofolate reductase* (*MTHFR*; rs1801131 and rs1801133). All of the SNPs were selected according to the following criteria: (1) the minor allele frequency was >5% in the Chinese population based on the HapMap HCB data; (2) the variant was located in the functional regions of the gene; (3) previously common, well studied variants. All researchers responsible for genotyping were blind to the clinical outcome. Detailed information of the five SNPs was shown in Table 1.

**Table 1:** Characteristics of the five SNPs.

SNP ID	Chromosome	Allele	Function consequence
*GSTP1*			
rs1695	11	A/G	Missense
*SOD2*			
rs4880	6	A/G	Missense
*GSTA1*			
rs3957356	6	C/T	Upstream variant 2KB
			Utr variant 5 prime
*MTHFR*			
rs1801131	1	G/T	Missense
rs1801133	1	A/G	Missense

### Statistical analysis

The development of ≥ 2 RP was defined as the end point in this study. The time to the end point was calculated from the start of RT. All analyses were performed with SPSS 17.0 (SPSS Inc., Chicago, IL). Hazard ratios (HRs) and 95% confidence intervals (CIs) were calculated by the Cox proportional hazards model. In addition, a multivariate Cox regression analysis was performed to adjust for other covariates. Kaplan–Meier analysis was used to evaluate the cumulative RP probability. All statistical tests were carried out with a two-sided P<0.05 level.

## Results

### Clinical characteristics and RP

The present study included a total of 265 ESCC patients (207 males and 58 females), with a mean age of 65 years (range from 18 to 91) (Table 2). All patients were part of the Chinese Han population. Among the 265 patients, 30.6% had pulmonary emphysema, 22.6% had hypertension, 6.8% had diabetes, and 72.1% were treated with a combination of chemotherapy and RT. The receiver operating characteristic (ROC) curve was used to determine the best cut-off values for RT dose, mean lung dose (MLD) and volume of normal lungs receiving 20 Gy or more radiation (V20) (55.7 Gy, 11.6 Gy, and 17.1%, respectively). Detailed dosimetric parameters for the whole population were as follows, total dose (range:50-70Gy, 95%CI: 50.00–67.38Gy), V20 (range:3–35%, 95%CI:6.70–29.97%) and MLD (range:3.39–23.70Gy, 95%CI: 5.27–17.78Gy). At the time of final analysis, the mean follow up time was 15.4 months (range: 1.3 to 24 months). Of the 265 patients, 42 (15.8%) developed ≥ 2 RP, and the mean occurrence time for ≥ 2 RP was 3.7 months.

**Table 2:** Patients demographics and associations with the risk of developing ≥ 2 RP (n = 265).

		Univariate analysis			Multivariate analysis		
Parameters	No. of patients (%)	HR	95% CI	P value	HR	95% CI	P value^a^
Age, years							
>65	127(47.9)	1			1		
≤65	138(52.1)	1.02	0.557–1.869	0.949	0.824	0.402–1.691	0.598
Gender							
male	207(78.1)	1			1		
female	58(21.9)	0.588	0.248–1.395	0.228	0.615	0.225–1.671	0.341
Smoking							
never	110(41.5)	1			1		
CSI≤600	81(30.6)	0.712	0.352–1.140	0.344	1.158	0.522–2.569	0.719
CSI>600	74(27.9)	0.660	0.303–1.436	0.295	0.664	0.287–1.536	0.339
Pulmonary emphysema							
No	184(69.4)	1			1		
Yes	81(30.6)	0.790	0.397–1.572	0.502	0.725	0.348–1.509	0.390
Diabetes							
No	247(93.2)	1			1		
Yes	18(6.8)	0.314	0.043–2.284	0.253	0.422	0.056–3.211	0.405
Hypertension							
No	205(77.4)	1			1		
Yes	60(22.6)	0.663	0.294–1.492	0.321	0.830	0.360–1.916	0.663
KPS							
>80	117(44.2)	1			1		
≤80	148(55.8)	1.320	0.708–2.460	0.383	1.412	0.731–2.728	0.305
Tumor location							
Cervical+ Upper thoracic	106(40.0)	1			1		
Middle thoracic	120(45.3)	0.637	0.294–1.381	0.253	0.689	0.301–1.574	0.376
Lower thoracic	39(14.7)	0.438	0.195–0.987	**0.046**	0.470	0.200–1.104	0.083
Stage							
II	58(21.9)	1			1		
III	185(69.8)	0.303	0.102–0.901	**0.032**	0.400	0.125–1.281	0.123
IV	22(8.3)	0.459	0.201–1.048	0.065	0.711	0.286–1.769	0.463
Chemotherapy							
No	74(27.9)	1			1		
Yes	191(72.1)	2.434	1.026–5.778	**0.044**	2.190	0.843–5.688	0.107
Radiotherapy							
CRT	120(45.3)	1			1		
IMRT	145(54.7)	0.615	0.334–1.134	0.119	0.708	0.367–1.366	0.303
Radiation dose, Gy							
>55.7	191(72.1)	1			1		
≤55.7	74(27.9)	0.491	0.218–1.107	0.086	0.557	0.240–1.294	0.173
MLD^b^, Gy							
>11.6	126(47.5)	1			1		
≤11.6	139(52.5)	0.186	0.086–0.403	**<0.001**	0.205	0.092–0.455	**<0.001**
V20^b^, %							
>17.1	154(58.1)	1			1		
≤17.1	111(41.9)	0.253	0.112–0.570	**0.001**	0.261	0.115–0.594	**0.001**

^a^ Multivariate analyses were adjusted for all of the factors in this table.

^b^ Either MLD or V20 was used in multivariate analyses, but not together.

Abbreviations: CSI, cigarette smoking index; KPS, Kamofsky performance status; CRT, conformal radiation therapy; IMRT, intensity-modulated radiation therapy; MLD, mean lung dose; V20, volume of normal lungs receiving 20 Gy or more radiation.

Table 2 also shows the association between patient-, tumor-, and treatment-related parameters and the risk of developing ≥ 2 RP. In the present study, MLD and V20 were found to be significantly associated with ≥ 2 RP in both univariate and multivariate analyses (MLD: HR = 0.186, 95% CI = 0.086–0.403, P<0.001, HR = 0.205, 95% CI = 0.092–0.455, P<0.001; V20: HR = 0.253, 95% CI = 0.112–0.570, P = 0.001, HR = 0.261, 95% CI = 0.115–0.594, P = 0.001,respectively). Tumor location, stage, and chemotherapy were also found to be statistically significant in univariate analyses (HR = 0.438, 95% CI = 0.195–0.987, P = 0.046; HR = 0.303, 95% CI = 0.102–0.901, P = 0.032; HR = 2.434, 95% CI = 1.026–5.778, P = 0.044, respectively); however, this differences were not significant in multivariate analyses.

### Genetic variants and RP

Table 3 shows the results of multivariate analysis of the association between genotypes and ≥ 2 RP by the Cox proportional hazards regression method. We found that rs1801131 of *MTHFR* was significantly related to the risk of ≥ 2 RP (HR = 0.285, 95% CI = 0.120–0.676, P = 0.004). Similar results were also observed in multivariate analyses, after adjusting for tumor location, stage, chemotherapy and V20. (HR = 0.339, 95% CI = 0.137–0.839, P = 0.019). The other genotypes had no significant associations with the risk of ≥ 2 RP in this study.

**Table 3:** Associations between genotypes and the risk of developing ≥ 2 RP.

			Multivariate analysis			Multivariate analysis		
Genotypes	No. of patients	No. of events	HR	95% CI	P^a^ value	HR	95% CI	P^b^ value
*GSTP1*:rs1695								
AA	155	27	1			1		
GG+GA	110	15	0.796	0.421–1.505	0.483	0.742	0.382–1.442	0.379
*SOD2*:rs4880								
AA	200	33	1			1		
GG+AG	65	9	0.888	0.424–1.862	0.753	1.068	0.503–2.268	0.864
*GSTA1*:rs3957356								
CC	196	36	1			1		
TT+CT	69	6	0.488	0.205–1.163	0.105	0.499	0.206–1.209	0.123
*MTHFR*:rs1801131								
TT	171	36	1			1		
GG+GT	94	6	0.272	0.120–0.656	**0.004**	0.339	0.137–0.839	**0.019**
*MTHFR*:rs1801133								
AA	90	15	1			1		
GG+GA	175	27	1.241	0.652–2.362	0.510	1.265	0.654–2.450	0.485

P^a^ values were calculated by Cox proportional model using multivariate analysis.

P^b^ values were calculated with adjustment for tumor location, stage, chemotherapy and V20.

**Fig 1:**
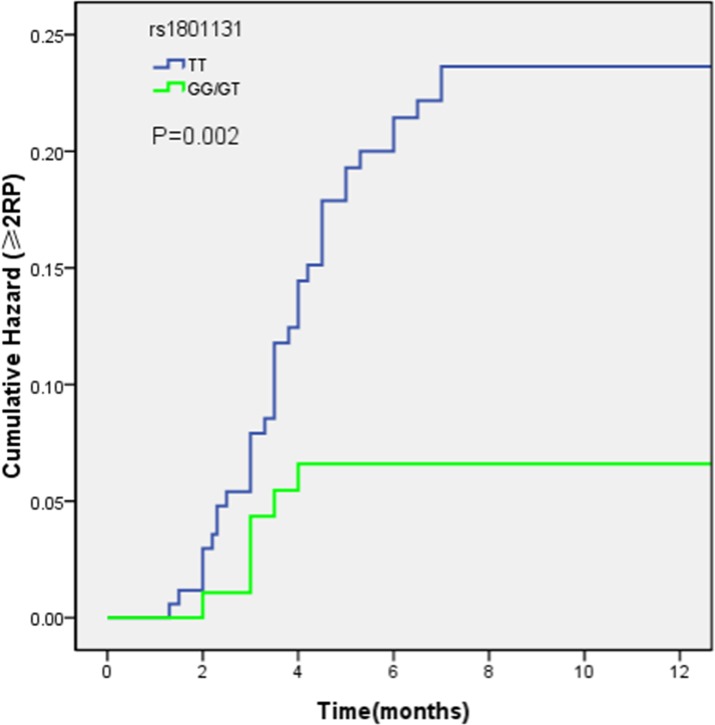
Kaplan-Meier estimated cumulative RP probability as a function of time from the start of RT by genotype of rs1801131. GG/GT genotypes was associated with a lower risk of ≥ 2 RP (P = 0.002).

In addition, cumulative RP probability for ≥ 2 RP of rs1801131 was calculated by Kaplan-Meier analysis. Generally, RP developed more often in patients exhibiting TT genotypes, with ≥ 2 RP rates of 19.7%, compared to those possessing GG+GT genotypes, for which the RP incidence was 6.7% (P = 0.002, Fig 1).

### *MTHFR*: rs1801131 and dosimetric parameters

**Fig 2:**
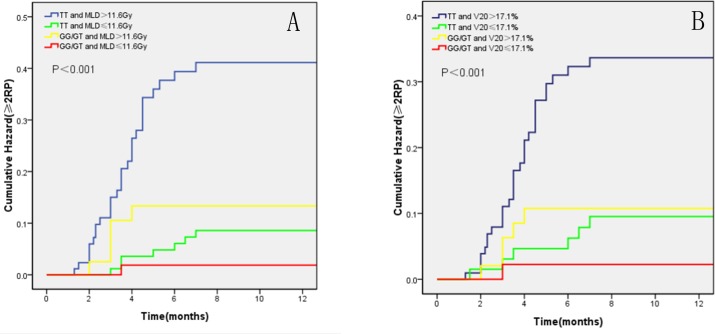
Kaplan-Meier estimated the effect of genotype of rs1801131 and dosimetric parameters on cumulative RP probability. (A) Patients with TT genotype of rs1801131 and MLD >11.6Gy had higher risk of ≥ 2 RP compared with GG/GT genotypes (P<0.001). (B) Patients with TT genotype of rs1801131 and V20 >17.1% had higher risk of ≥ 2 RP compared with GG/GT genotypes(P<0.001).

Since dosimetric parameters were also risk factors for RP, we further performed subgroup analysis to determine whether the rs1801131genotype is independent from dosimetric factors. Patients were divided into four groups according to genotype in rs1801131 and MLD or V20. Patients with TT genotypes in rs1801131 and MLD>11.6 Gy or V20>17.1% had the highest RP risk compared with other groups (P<0.001; P<0.001,respectively,Fig 2). However, this difference was not significant in patients who received MLD ≤ 11.6 Gy or V20 ≤ 17.1%, suggesting that the effect of genetic variants on the risk of ≥ 2 RP were independent from dosimetric parameters.

## Discussion

In our prospective study, we examined the association between genetic variants in *SOD2*, *GSTP1*, *GSTA1 and MTHFR* genes and the RP risk in ESCC patients. We found that rs1801131 of *MTHFR* gene in the DNA repair pathway maybe a reliable and independent predictor for ≥ 2 RP. The study indicated that patients with GG/GT genotypes had a decreased risk of ≥ 2 RP compared with other genotype. The effect of this genotype on the risk of RP was independent from dosimetric factors. However, genetic variants in ROS metabolism-related genes were not found to be significantly associated with RP risk.

**Fig 3:**
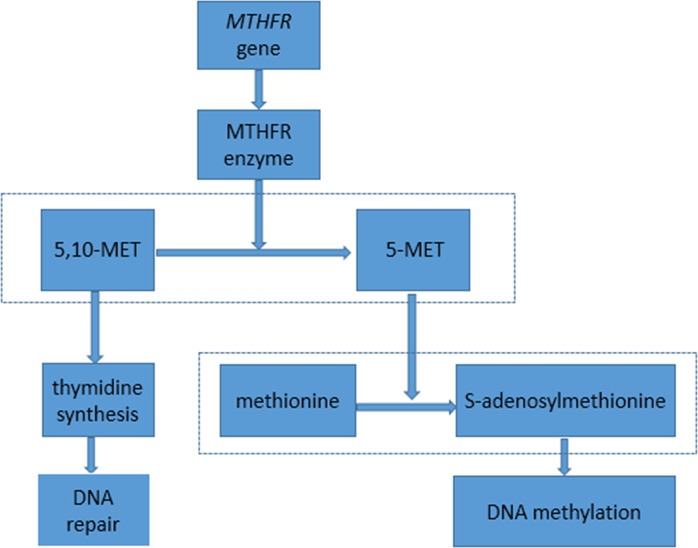
Information about the *MTHFR* gene participating in the repair of DNA. Abbreviations: 5,10-MET: 5,10-methylenetetrahydrofolate; 5-MET: 5-methylenetetrahydrofolate.

MTHFR is an important enzyme, that catalyze the irreversible conversion of 5,10-methylenetetrahydrofolate to 5-methyltetrahydrofolate. The latter is the main form of circulatory folate, which converts methionine to S-adenosylmethionine, and S-adenosylmethionine is the universal methyl donor for various intracellular methylation reactions, particularly DNA methylation [18–21]. The former 5,10 methylenetetrahydrofolate, participating in the synthesis of thymidine, is believed to be critical in the repair of DNA [14]. Detailed information was shown in Fig 3. The MTHFR enzyme is encoded by the *MTHFR gene*, which is localized to chromosome 1p36.3 spanning over 20 kb and contains a noncoding exon (exon 1) and 11 coding exons [22]. The rs1801131 of *MTHFR* results in a glutamate to alanine substitution at codon 439 in exon 7, which is located in the COOH-terminal regulatory domain of the gene, and results in a 30–40% reduction of enzymatic function in the homozygous variant genotype [22]. What’s more, other research demonstrated that after total body irradiation in mice, MTHFR activity levels decreased while thymidylate synthase activity increased suggesting that the regulation of these enzymes in diverting folate metabolism toward thymidine base synthesis may play an important role in the cellular response to ionizing radiation [23].In addition, recent investigations have found that genetic variants of *MTHFR* were related to many diseases [24–29].

A further study conducted by Harvard School of Public Health found that genetic variants in *MTHFR* gene associated significantly with RP risk for lung cancer patients receiving radiotherapy in white population [17]. Although well designed, this research still has several shortcomings, which deserves more heed. Firstly, the lung caner patients in this article were retrospectively collected from 2001 to 2009. It is notoriously difficult to accurately identify and grade radiation pneumonitis just based on medical records. Then, the whole population of the research was relatively inhomogeneous for including both small cell and non-small cell lung cancer patients. Furthermore, the sample set was relatively small, which only constituted by 136 patients. In addition, it is well known that frequencies of SNPs vary significantly between different ancestries, which further influence disease susceptibility. It is also unclear whether this positive finding still showns influence in other groups. Taken all issues above into consideration, we performed this prospective study in a relatively large Chinese Han ESCC population.

Dynamic interactions involving radiation-induced death of target cells, and generation of ROS are believed to contribute to radiation tissue injury [30]. Indirect damage caused by ROS commonly plays a bigger role in the process of RP. Recent studies also illustrated genetic variants involved in ROS metabolism pathway-related genes could predict radiation-induced skin toxicity for breast cancer patients receiving RT [31]. However, in our analysis, while CC genotype of rs3957356 in *GSTA1* gene shows a relative high risk of RP, the results was still not statistically significant, which further studies are deserved to confirm the results.

Our study has several advantages. Firstly, as is well established, RP is somewhat a diagnosis of exclusion, it is very difficult to accurately assess and grade radiation toxicity based on medical records in retrospective studies [8]. Thus, our prospective study has the advantage of objective and accurate diagnosis of RP. Another strength of our study is relative uniform population, which is achieved by including only Chinese Han patients with locally advanced ESCC to eliminate the influence of confounding variables.

However, our study is also limited by some shortcomings. Firstly, we have a relative small sample size, thus our findings should be validated in a larger and multicenter population. Secondly, the genetic variants included in this study were candidate SNPs based on known or predicted effects on gene function. A candidate-gene approach has the advantage of being anchored by known biological plausibility, but there is a possibility that this study has missed additional risk alleles or detected a variant in linkage disequilibrium with the true causative SNPs [32]. Thirdly, as some genetic markers are ethnic specific, our results also should be validated among different ethnic populations in the future. In addition, the functional SNPs in the *MTHFR* gene are significantly associated with RP, but the exact mechanism by which this genetic variant alters the risk of RP, is still unknown, and therefore warrants further study.

In conclusion, our study explored the association between genetic variants in candidate genes involved in ROS metabolism, DNA repair pathways and the risk of RP. We identified that rs1801131 of *MTHFR* gene in DNA repair pathway was statistically associated with ≥ 2 RP. However, our conclusion should be interpreted with caution in clinical practice because of several problems encountered in this field [33–35]. Further validation studies and investigations into the underlying mechanisms involving the genetic variants are required.
